# Semiparametric models for malaria rapid diagnosis test result

**DOI:** 10.1186/1471-2458-14-31

**Published:** 2014-01-13

**Authors:** Dawit G Ayele, Temesgen T Zewotir, Henry G Mwambi

**Affiliations:** 1 School of Mathematics, Statistics and Computer Science, University of KwaZulu-Natal, Pietermaritzburg, Private Bag X01, Scottsville 3209, South Africa

**Keywords:** AM, GAMM, Rapid diagnostic test

## Abstract

**Background:**

More than 75% of the total of Ethiopia is malarious. Therefore, malaria is a leading public health problem in Ethiopia. This study aims to identify socio-economic, geographic and demographic factors contributing to the spread of malaria and is based on the results of a malaria Rapid Diagnosis Test survey.

**Methods:**

The data used in this study originates from the baseline malaria indicator survey, conducted in the Amhara, Oromiya and Southern Nation Nationalities and People (SNNP) regions of Ethiopia from December 2006 to January 2007. The study applies the method of generalized additive mixed model (GAMM) to analyse data. The response variable is the presence or absence of malaria, using the malaria Rapid Diagnosis Test (RDT).

**Results:**

The results provide an improved insight into the distribution of malaria in relation to the age of affected people, the altitude, the total number of rooms, the total number of mosquito nets, family size, and the number of months that their rooms have been sprayed. The results confirm that positive malaria RDT test results are high for children under 15 years and for older persons. Gender, source of drinking water, time needed to fetch water, toilet facilities, main materials used for the construction of walls, floors and roofs, and use of mosquito nets were all found to have a significant impact on the results of the malaria rapid diagnosis test.

**Conclusion:**

The result of the analysis identifies poor socio-economic conditions as a major contributing factor or determinant for the spread of malaria. With the correct use of mosquito nets, indoor residual spraying with insecticide and other preventative measures, the incidence of malaria could be decreased. In addition, improving housing conditions is a means to reduce the risk of malaria. Other measures such as creating awareness of the use of mosquito nets, indoor residual spraying with insecticide, and malaria transmission, can lead to a further reduction in the number of malaria cases.

## Background

Malaria is a life-threatening disease affecting the world’s most under-developed countries and regions as well some developed countries [1]. In Sub-Saharan Africa, malaria is a major cause of morbidity and mortality [1]. It is a dominant public health problem in Ethiopia and since many years the prime cause of illness and death in that country [2,3]. Epidemics of malaria are relatively frequent [4,5], involving highland or highland fringe parts of Ethiopia, mainly those areas that are 1,000 to 2,000 meters above sea level [6-8]. To control the risk of malaria, early diagnosis and prompt treatment are key strategies. Diagnosing malaria is mostly done by clinical diagnosis, but laboratory facilities are not available everywhere in the country [9,10]. The standard method for diagnosing malaria is through microscopy. However, this form of diagnosis is not available or affordable in most of the peripheral health facilities. The recent introduction of RDT for malaria is a significant step forward as regards detection, timely treatment and management of the disease as well as reduction of unnecessary treatment. Malaria RDT can be applied in malaria diagnosis during population-based surveys and, on the basis of the results, treatment can begin promptly.

RDTs for malaria offer the potential to extend the provision of accurate malaria diagnosis to areas where microscopy services are not available, for example in remote locations or after regular laboratory hours. Rapid malaria diagnostic tests have been developed in the lateral flow format [11]. These tests use finger-stick or venous blood and take only 10 to 15 minutes to complete without requiring laboratory involvement. Non-clinical staff can easily learn to perform the test and to interpret the results [12].

The identification of socio-economic, demographic and geographic risk factors associated with the prevalence of malaria by using data, obtained from the rapid diagnosis test, is an important exercise. Such a study is helpful in determining which households are in critical need of intervention. Therefore, the aim of this study is to determine the socio-economic, demographic and geographic risk factors for being affected by malaria, on the basis of the results of the RDT survey as analysed using novel and flexible statistical methods.

## Methods

### Study design

From December 2006 to January 2007, The Carter Center (TCC) in Ethiopia has conducted a baseline household cluster malaria survey. A relevant questionnaire was developed as a modification of the Malaria Indicator Survey (MIS) Household Questionnaire. It consists of two parts, a household interview and a malaria parasite form. The sampling frame for the survey among the rural populations of the Amhara, Oromiya and SNNP regions consisted in the case of each in a *Kebele* (the smallest administrative unit in Ethiopia). From the three regions 5,708 households in total, located in 224 clusters, were included in the survey. Of the total number of 5,708, in the Amhara, Oromiya and SNNP regions 4,101 (71.85%), 809 (14.17%), and 798 (13.98%) households were covered respectively. Prior to the survey, 224 *Kebeles* were selected. From each *Kebele*, 12 households (even-numbered) were selected for malaria tests. For survey purposes, each room in the participants’ dwellings was listed separately. Using the amount of mosquito nets present, it was possible to ascertain the number of occupants per room as well as how many rooms, both inside and outside each dwelling, were used as bedrooms. In addition to the number of rooms and the number of nets, the number of persons sleeping under each net was listed. The detailed sampling procedure is presented in [13-15].

Before testing them for malarial parasites, consent was obtained from the participants. To collect samples, finger-prick blood was collected from each participant for the malaria rapid diagnostic test. The test used is known as *ParaScreen* and is capable of detecting Plasmodium *falciparum* as well as other *Plasmodium* species. Participants, whose positive rapid tests, were immediately offered treatment according to national guidelines.

### Variables of interest

#### Response variable

The outcome of interest is the malaria RDT result. Malaria RDTs assist in the diagnosis of malaria by detecting evidence of malaria parasites in human blood. They are an alternative to diagnosis based on clinical grounds or on microscopy, particularly where good quality microscopy services cannot be readily provided. Thus, the response variable is binary, indicating whether or not a person is positive for malaria, using the malaria RDT.

### Independent variables

The independent variables or covariates are the baseline socio-economic, demographic and geographic variables. These variables were collected at household and individual levels. The malaria test (RDT) result and facts concerning age and sex of participants were collected at individual level. Other information - regarding the altitude at which participants live, their main source of drinking water, the time taken to collect water, their toilet facilities, availability of electricity, radio, television, the total number of rooms they inhabit, the main material of the walls, roof and floors of the rooms, the question whether indoor residual spraying (IRS) with insecticide has taken place in the past 12 months, the use of mosquito nets and the total number of nets in their dwelling – was collected at household level.

### Statistical methods

In previous studies, parametric methods were used to fit the malaria RDT result data [13,16,17]. These models provide a powerful tool for modelling the relationship between a response variable and covariates. In many applications, the functional form of the relationship may only be partly specified. The relationships between the response and some confounding covariates may have unknown functional form. This leads to the study of semiparametric additive models. Generalized Additive Models (GAM) which was proposed by Hastie and Tibshirani in 1986, generalizes by adding a parametric nonlinear component to the additive predictor on the link scale [18-20]. This type of model structure has wide applications in scientific studies where some parametric nonlinear regression relationship is of main interest. Using parametric methods might have confounding effects for some covariates whose relationship to the response is of unknown functional form. Therefore, in such kind of parameters, it is important to estimate nonparametrically. Therefore, nonparametric smoothing methods are applied in statistics as a flexible tool in finding structure and connections within data. Furthermore, GAM extended from Generalized linear model replacing the linear form with the additive form, i.e., ∑ _*i*_*X*_*i*_*β*_*i*_ and ∑ _*i*_*f* (*X*_*i*_) respectively. To determine the appropriate smooth function *f*, the steps in GLM replaced by nonparametric additive regression steps. Therefore, the GAM can be presented as [20].

(1) $$g\left(\mu_{i}\right)=X_{i}^{*}\theta +f_{1}\left(x_{1i}\right)+f_{2}\left(x_{2i}\right)+f_{3}\left(x_{3i}\right)+\ldots +f_{p}\left(x_{\mathrm{pi}}\right)$$

where *μ*_*i*_ ≡ *E*(*Y*_*i*_) and *Y*_*i*_ distributed some exponential family distribution, *X*_i_^*^ is the design matrix, *θ* is the corresponding parameter vector, and *f*_*j*_(.) are smooth functions of covariates. Model (1) is simply an additive model if *g* is the identity link and the response is normally distributed. Moreover, estimation of parameters for GAM depends on the choice of smoothing bases. Scatterplot smoothing functions, commonly referred to as smoothers, are central to GAM. A smoother is a tool used for summarizing the trend of a response measurement as a function of independent variables [19].

For data which consists repeated measurement or other correlations, the correlations introduce a new source of randomness and create an extension to GAM. Similar to generalized linear mixed models (GLMM) are extensions of GLM, generalized additive mixed models (GAMM) are extensions of GAM and allows the parametric fixed effects to be modelled nonparametically using additive smooth functions. Therefore, GAMM’s include random effects and has the following structure [19,21,22].

(2) $$g\left(\mu_{i}\right)=\beta_{0}+f_{1}\left(x_{i1}\right)+\ldots +f_{p}\left(x_{\mathrm{ip}}\right)+Z_{i}b$$

where, *y*_*i*_, *i* = 1, …, *n* is outcome variable, *p* covariates *X*_*i*_ = (1, *x*_*i*1_, …, *x*_*ip*_)^’^ associated with fixed effects and *q* × 1 vector of covariates *Z*_*i*_ associated with random effects. Therefore, given a *q* × 1 vector of ***b*** of random effects, the observations *y*_*i*_ are assumed to be conditionally independent with means *E*(*y*_*i*_|*b*) = *μ*_*i*_ and variances, *var* (*y*_*i*_| ***b***) = Ø *v*(*μ*_*i*_) where *v*(.) is a specified variance function and Ø is a scale parameter. Moreover, *g*(.) is a monotonic differential link function. *f*_*i*_(.) is a centred twice-differentiable smooth function and the random effects are assumed to be distributed as *N*{0, *G*(*γ*)} and *y* is a *c* × 1 vector of variance components. To model correlations between observations, the additive nonparametrics are used [23].

For a given variance component, *θ* the log-quasi-likelihood function of, {*β*, *f*_*i*_, *θ*} a part from a constant

(3) $$\begin{array}{l} \exp[l\{y;\beta_{0},f_{1}\left(.\right),\ldots ,f_{p}\left(.\right),\theta ]\propto {\left|G\right|}^{-1/2}\\ \;\;\int \exp\left\{-\frac{1}{2Ø}\sum_{i=1}^{n}d_{i}\left(y;\mu_{i}\right)-\frac{1}{2}b^{'}G^{-1}b\right\}db \end{array}$$

where

*y*_*i*_ = (*y*_1_, …, *y*_*n*_)^'^ and $d_{i}\left(y;\mu \right)\propto -2\int_{y_{i}}^{\mu_{i}}\frac{y_{i}-u}{\nu \left(u\right)\mathrm{du}}$ defines the conditional deviance function of {*β*, *f*_*i*_, *θ*} given ***b***.

The estimation of smooth parameters, λ, and inference on variance component *θ* is required for GAMM statistical inference on the nonparametric functions *f*_*j*_(.). It has to be noted that smoothing spline estimators and linear mixed models have close connections [22,24,25]. As explained in Green *et al*. [26], for a given value of λ and *θ*, the natural cubic smoothing spline estimators of *f*_*i*_(.) maximize the penalized log quasi-likelihood.

(4) $$\begin{array}{l} l\left\{y;\beta_{0},f_{i}\left(.\right),\theta \right\}-\frac{1}{2}\sum_{j=1}^{p}\lambda_{j}\int_{s_{j}}^{l_{j}}f_{j}^{''}{\left(x\right)}^{2}\mathrm{dx}\\ \;=l\left\{y_{i};\beta_{0},f_{i}\left(.\right),\theta \right\}-\frac{1}{2}\sum_{j=1}^{p}\lambda_{j}f_{j}^{'}S_{jf_{j}} \end{array}$$

where (*S*_*j*_, *t*_*j*_) defines the range of the *j*^*th*^ covariate and *λ* = (*λ*_1_, …, *λ*_*p*_)^'^ is a vector of smoothing parameters. The trade-off between goodness of fit and the smoothness of the estimated functions is controlled by *λ.* Furthermore, *f*_*j*_(.) is an *r*_*j*_ × 1 unknown vector of the values of *f*_*j*_(.) evaluated at the *r*_*j*_ ordered values of the *x*_*ij*_ = (*i* = 1, …, *n*) and *S*_*j*_ is the smoothing matrix.

Using the matrix notation the GAMM model, which is given in (2), can be written as

(5) $$g\left(\mu_{i}\right)=1\beta_{0}+N_{1}f_{1}+\ldots +N_{p}f_{p}+\mathrm{Zb}$$

Where *g*(*μ*_*i*_) = {*g*(*μ*_1_), …, *g*(*μ*_*n*_)}^'^,  and ***Z*** =  (*Z*_1_, …, *Z*_*n*_)^'^

The numerical integration is required to evaluate the expression given in (3). To calculate full natural cubic smoothing spline estimators of *f*_*j*_ by directly maximizing (4) is sometimes difficult. Therefore, to avoid this problem an alternative approximation proposed by Lin and Zhang [22]. This proposed method is a double penalized quasi-likelihood (DPQL). Therefore, the nonparametric functions (*f*_*j*_ ) estimation can be obtained by using double quasi-likelihood. Here, *f*_*j*_  (centered parameter vector) can be re-parametrized in terms of *β*_*j*_ and *a*_*j*_((*r*_*j*_ − 2) × 1) through a one-to-one transformation as

(6) $$f_{j}=X_{j}^{*}\beta_{j}+B_{j}a_{j}$$

where *X*_j_^*^ is *r*_*j*_ × 1 vector containing the *r*_*j*_ centered district values of the, *x*_*ij*_ (*i* = 1, …, *n*) and *B*_*j*_ = *L*_*j*_(*L*_*j*_^’^*L*_*j*_)^−1^ and *L*_*j*_ is an *r*_*j*_ × (*r*_*j*_ − 2) fullrank matrix satisfying *S*_*j*_ = *L*_*j*_*L*_*j*_^’^ and *L*_*j*_^’^*x*_j_^*^ = 0 Therefore, the double penalized quasi-likelihood with respect to (*β*_0_, *f*_*i*_) and ***b*** becomes.

(7) $$-\frac{1}{2Ø}\sum_{i=1}^{n}d_{i}\;\left(y;\mu_{i}\right)-\frac{1}{2}b^{'}G^{-1}b-\frac{1}{2}{a^{'}}^{-1}a,$$

where *f*_*j*_^’^*S*_*j*_*f*_*j*_ = *a*_*j*_^’^ *a*_*j*_$,a=\left(a_{1}^{'},\ldots ,a_{p}^{'}\right)\;\mathrm{and}=\mathrm{diag}\left(\tau_{1}I,\ldots {,}_{p}I\right)\;\mathrm{with}\;\tau_{j}=1/\lambda_{j}$ Note that small values of *τ* = (*τ*_1_, …, *τ*_*p*_)^'^ corresponds to oversmoothing [21].

## Result

In previous studies, the malaria RDT result had been fitted to predictor variables, using parametric models whereby a linear age, family size, number of rooms per person, number of nets per person, altitude of dwellings, and number of months since rooms had been sprayed, were based on the assumption of having linear relation with malaria RDT result [13,16,17]. However, the objective of the study under discussion was to model the effect of age, family size, number of rooms per person, number of nets per person, altitude and number of months since spraying the rooms non-parametrically, while the other covariates remain parametric using GAMM. Because these factors have continuous effect, they could have non-linear relationships with malaria RDT. Therefore, fitting these variables non-parametrically is important. The final GAMM model consists of the following socio-economic, demographic and geographic factors: gender, age, family size, region, altitude, main source of drinking water, time taken to collect water, toilet facilities, availability of electricity, radio and television, total number of rooms, main material of the rooms’ walls, main material of the roofs, main material of the floors, indoor residual spraying (IRS) in the past 12 months, use of mosquito nets and total number of nets, malaria test (RDT result). Therefore, the malaria RDT result with the semi-parametric logistic regression model was fitted with all these variables, including possible interaction effects. Unlike in previous models [13,16,17], the factors age, family size, number of rooms per person, number of nets per person, altitude and number of months since the rooms were last sprayed, were fitted non-parametically. Therefore, the final model is given as follows.

(8) gμij=β0+β1Genderi+β2Region2+β3drinking_wateri+β4time_to_get_wateri+β5toilet_facilityi+β6electi+β7tvi+β8radioi+β9room_wallroom_roofi+β10room_walli+β11indoorresidualsprayingwithinsecticidei+β12net_usei+β13Gender*drinking_wateri+β14Gender*electi+β15Gender*room_walli+f1agei+f2altitudei+f3famsizei+f4total_roomi+f5total_netsi+f6months_sprayedi+b0i

where *g*(.) is the logit link function, *β’s* are parametric regression coefficients, *f*_*j*_’s are centred smooth functions and the random effects, *b*_*i*_ ~ *N*(0, *G*(*θ*)). Therefore, the estimation procedures discussed for fitting GAMMs in the previous section can be used to fit model (8). For the analysis, R package (*mgcv*) was used. There are many smoothing spline options in R package. Among the number of options to fit model (8), several different penalized regression smoothers were used. Because of the size of the model and the size of the dataset, the model failed to converge for more interaction effects. Model (8) contains reduced parameters by removing the three-way parametric interactions, i.e., important effects are included.

Thin plate shrinkage smoothers were used to fit model (8). The use of shrinkage smoothers has several advantages, i.e., they help to avoid the knot placement. Furthermore, the methods can be constructed to smooth of any number of predictor variables. Construction of shrinkage smoothers depends on the smooth terms which can be penalized away. This makes no contribution to the model [20].

Table 1 presents the significant effects for the parametric coefficients of the model. The table shows that gender, region, main source of drinking water, time spent to get water, toilet facilities, availability of electricity, availability of radio, main materials used for the construction of walls, roofs, and floors of rooms, effect of indoor residual spraying with insecticide and the use of mosquito nets, were found to have significant consequences for the malaria rapid diagnosis test result. The factors of gender, main source of drinking water, availability of electricity, main materials for the construction of walls and roof of the participant’s room, were all involved in the interaction effects. These interaction effects concern gender and main source of drinking water, gender and availability of electricity, gender and main material of the walls of one’s room, and main source of drinking water and main material of the roof of one’s room (Tables 1 and 2).

**Table 1:** The parameter estimates of the GAMM model of the main parametric coefficients

**Parameter**	**OR**	**95% CI**		**Pr > |t|**
		**Lower**	**Upper**	
Intercept	0.047	0.032	0.077	<.0001
Sex (ref. Male)				
Female	0.179	0.109	0.256	<.0001
Region (ref . SNNP)				
Amhara	0.969	0.728	0.906	0.7041
Oromiya	0.807	0.594	0.832	0.0225
Main source of drinking water (ref. protected water)				
Protected water	0.899	0.978	1.047	0.1744
Tap water	1.795	0.647	1.917	<.0001
Time to collect water (ref. greater than 90 minutes)				
Between 30 and 40 minutes	0.291	0.232	0.454	<.0001
40 - 90 minutes	0.293	0.230	0.456	<.0001
Greater than 30 minutes	0.361	0.298	0.524	0.0024
Toilet facility (Ref. No facility)				
Pit latrine	0.656	0.593	0.819	0.0057
Toilet with flush	0.500	0.463	0.663	<.0001
Availability of electricity (ref. no)				
Yes	1.117	1.054	1.280	<.0001
Availability of television (ref. no)				
Yes	1.050	1.087	1.213	0.383
Availability of radio (ref. no)				
Yes	2.158	2.095	2.321	<.0001
Main material of room's wall (ref. cement block)				
Corrugated metal	0.333	0.326	0.496	<.0001
Mud block/stick/wood	0.427	0.364	0.590	<.0001
Main material of room's roof (ref. corrugate)				
Stick and mud	3.294	3.231	3.457	<.0001
Thatch	2.351	2.288	2.414	0.0002
Main material of room's floor (ref. earth/Local dung plaster)				
Wood	0.198	0.163	0.261	<.0001
Cement	0.052	0.015	0.115	<.0001
Anti-malarial spraying (ref. yes)				
No	3.438	3.375	3.601	<.0001
Use of mosquito nets (ref. no)				
Yes	0.506	0.443	0.669	<.0001

**Table 2:** The parameter estimates of the GAMM model of the interaction parametric coefficients

**Parameter**	**OR**	**95% CI**		**Pr > |t|**
		**Lower**	**Upper**	
Gender and main source of drinking water (ref. Male & protected water)				
Female and Tap water	5.795	5.732	5.958	<.0001
Female and Unprotected water	0.201	0.138	0.364	<.0001
Gender and availability of electricity (ref. Male & yes)				
Female and No	6.366	6.303	6.529	<.0001
Gender and main material of room's wall (ref. Male & earth/Local dung plaster)				
Female and wood	0.596	0.533	0.759	<.0001
Female and cement	0.010	0.053	0.173	0.888
Main source of drinking water and main material of the room's roof (ref. Protected water & thatch)				
Tap water and Mud block/stick/wood	0.024	0.019	0.187	<.0001
Tap water and Corrugated metal	0.021	0.018	0.184	0.006
Unprotected water and Mud block/stick/wood	0.018	0.014	0.181	<.0001
Unprotected water and corrugated metal	0.266	0.203	0.429	<.0001

The GAMM analysis shows that the odds of positive malaria RDT results for households living in the Amhara region were 0.969 (*e*^−0.031^) times lower than for inhabitants of the SNNP region. Similarly, the odds of a positive malaria RDT outcome for respondents who live in the Oromiya region were found to be 0.807 (*e*^−0.215^) times lower than for households in the SNNP region. Also, respondents who had to travel more than 40 minutes to fetch water were 0.361 (*e*^−1.019^) times less likely to test positive for malaria RDT than those who travel more than 90 minutes, followed by those who needed between 30 to 40 minutes (0.293 (*e*^−1.226^)) to get water, and those who travel less than 30 minutes (0.291 (*e*^−1.233^)). Similarly, the odds of positive malaria RDT results for respondents who use flush toilets were found to be 0.5 (*e*^−0.649^) times lower than for those who have no toilet facility, followed by owners of pit latrines (0.656 (*e*^−0.421^)). Another factor of the study was that households without access to radio were 2.158 (*e*^0.769^) times more likely to prove positive for malaria in an RDT test than those who do. Also, respondents who live in houses with cement floors were 0.052 (*e*^−2.957^) times less likely to test positive in the malaria RDT, compared to those whose dwellings have earth/local dung floors and followed by occupants of houses with wooden floors (0.198 (*e*^−1.621^)).

### Interaction effects

In addition to the main parametric effects, the fitted GAMM model contains four two-way interaction effects. These effects are gender and main source of drinking water, gender and availability of electricity, gender and main material of the walls of one’s room, and main source of drinking water and main material of the room's roof (Table 2).

**Figure 1:**
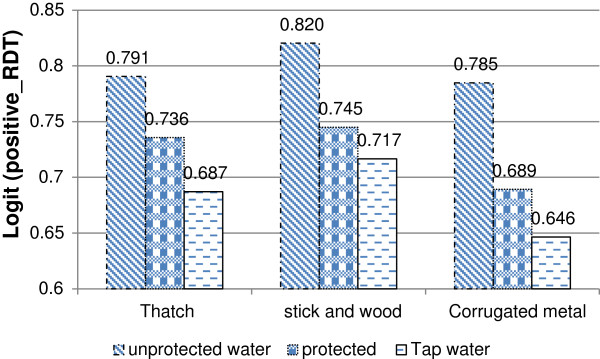
Log odds associated with rapid diagnosis test and source of drinking water with material of the room's roof.

Interaction effects between the main source of water and the main material used for the roof of one’s room is presented in Figure 1. From the figure, it is clear that the positive rapid diagnosis of malaria was significantly higher for households with a stick and mud roof than for those who have a thatch roof, followed by those who live under a corrugated iron roof. There were similar results in the case of respondents who use tap water in comparison to those who drink protected, and those who use unprotected water for drinking (Figure 1). Furthermore, there was a significant difference in the results of the rapid diagnosis test between the users of tap water, protected and unprotected sources of drinking water and were living under thatch and stick and mud roofs. The figure also shows significantly fewer respondents, living under corrugated iron roofs and using tap water for drinking, test positive for the malaria RDT than those who use protected and unprotected sources for drinking water. Unprotected water includes: unprotected springs, unprotected dug wells (using bucket and rope), and surface water (river/dam/lake/pond/stream). Protected water sources include: capped springs, protected dug wells (using a hand pump), tube wells or boreholes and carts with small tanks. Furthermore, tap water includes public taps or standpipes, piping water into one’s yard or dwelling.

**Figure 2:**
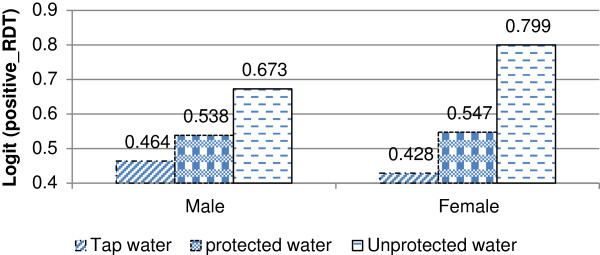
Log odds associated with rapid diagnosis test and main source of drinking water with gender.

Another significant two-way interaction effect occurs between gender and main source of drinking water (Table 2). The probability of a positive rapid diagnosis test was considerably higher among those female household members who use unprotected drinking water than for females who drink protected and tap water. Male households members are generally less likely to test positive for malaria than female household members (Figure 2). However, for female household members using tap water positive test results were lower compared to males.

**Figure 3:**
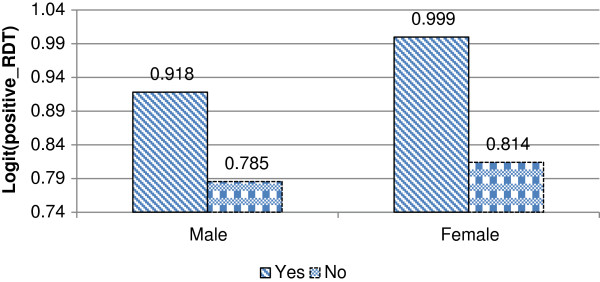
Log odds associated with rapid diagnosis test and availability of electricity with gender.

Figure 3 presents the interaction effect involving availability of electricity and individual gender. As the figure indicates, the prevalence of malaria was significantly higher for female than for male respondents who were living in a house with electricity. Similarly, females living in dwellings with electricity, showed higher positive malaria test results than males who have no electricity.

**Figure 4:**
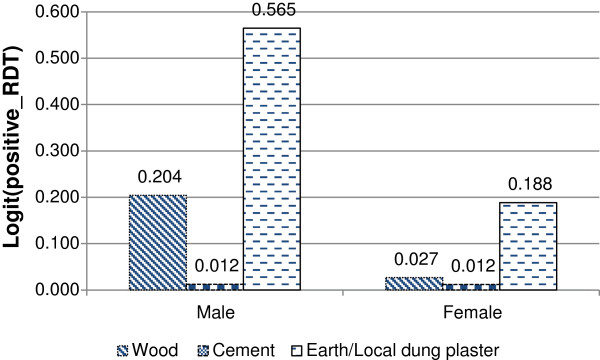
Log odds associated with rapid diagnosis test and main material of floor with gender.

The interaction effect between gender and the main material of floors is presented in Figure 4. The figure shows that the odds of positive RDT for male and female members of households living on earthen or /local dung floors, are significantly more likely to test positive for malaria than households whose dwellings have wooden and cement floors. For female members of the household the odds of testing positive for malaria RDT was higher when they were living on earthen or local dung floors.

**Figure 5:**
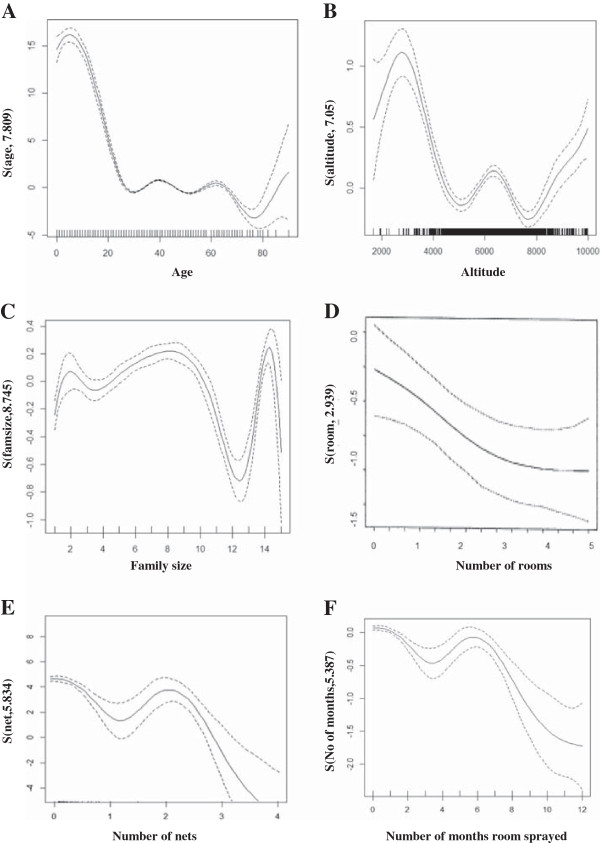
Smoothing components for malaria RDT with A) age, B) Altitude, C) Family size, D) Total number of rooms, E) Total number of nets and F) number of months room sprayed.

In addition to parametric effects, there are effects which were handled non-parametrically to the model. Age, altitude, family size, total number of rooms, total number of nets and the number of months since the room has been sprayed, have been fitted as a smooth. The result in Table 3 shows that age, altitude, family size, total number of rooms, total number of nets and number of months since the room was sprayed, have a significant impact on malaria RDT results. The smooth term for these effects has been presented in Figure 5. The figure suggests that the effects of the factors age, altitude, family size, total number of rooms, total number of nets and number of months since spraying the room, depart dramatically from linearity.

**Table 3:** Approximate significance of the smooth terms

**Source**	Edf*	**F-value**	**P-value**
S(age)	7.809	461.1	<.0001
S(altitude)	7.050	39.25	<.0001
S(family size)	8.745	25.07	<.0001
S(total number of room)	2.939	24.56	<.0001
S(total number of nets)	5.834	15.62	<.0001
S(number of month room sprayed)	5.387	16.01	<.0001

*Estimated degree of freedom.

Figure 5 gives the estimated smoothing components for malaria RDT result listed as A) age, B) altitude, C) family size, D) total number of rooms, E) total number of nets, F) number of month since room has been sprayed. In each panel, the smooth line represents the estimated trend of a generalized additive mixed model for the model with spherical Gaussian covariance structure. Figure 5a shows the estimated smooth function of age $\left(\overset{＾}{f}\left(\mathrm{age}\right)\right)$and its 95% confidence interval. The y-axis represents the effect of the age term, where *s* is a smoother term and the number in parentheses stands for the estimated degrees of freedom (edf). The figure suggests that participants’ malaria positive RDT results are higher at early age, increasing during the first five years and steadily decreasing afterwards. The test statistic is 461.1 with 7.809 degrees of freedom, providing strong evidence (p-value = <0.0001) against the assumption that age is linearly associated with malaria positive RDT results (Table 3). Figure 5b shows the estimated smooth function for the altitude at which participants live. Larger edfs value in the figure (7.05) corresponds to increasingly nonlinear relationships. Moreover, the malaria RDT results are higher for the first 3000 meters and start to decrease at greater altitudes. In addition to this, family size has a significant effect on malaria prevalence (Table 3). The estimated smooth function for family size is presented in Figure 5c. The figure shows that edf is 8.745, which indicates an increasingly nonlinear relationship. Moreover, the F-value is 25.07 with p-value < .0001, suggesting that family size is not linearly associated with malaria RDT test results. Other significant factors were found to be the total number of rooms occupied by a household, the total number of nets, and the number of months since a room was last sprayed indoors with insecticide. The estimated degrees of freedom are 2.939, 5.834 and 5.387 respectively. These figures suggested nonlinear relationships with malaria RDT results.

## Discussion

The prevalence of malaria is related to poor socio-economic conditions. It is usually referred to as a disease of poverty [27]. Malaria disproportionately affects poor people who cannot afford treatment or who have limited access to health care. Malaria-affected families and communities are often trapped in a downward spiral of poverty [28]. It is important to understand the links between the prevalence of malaria and the socio-economic conditions in a community. Identifying socio-economic factors that increase the risk of being affected by malaria can help to guide government policy-makers towards the creation and implementation of more effective policies to tackle the disease.

In previous studies, the malaria rapid diagnosis test data was investigated using the generalized linear model (survey logistic), generalized linear mixed models (GLMMs), and the spatial statistics method [13,16,17]. These methods were employed to fit the malaria RDT data. The models provide a powerful tool for modelling the relationship between a response variable and covariates. Such parametric mean models are simple to use. On the basis of several sophisticated applications, many computationally intensive data analytic modelling techniques have been invented. These are useful to explore possible hidden structures and to reduce modelling biases of the parametric methods. The restrictions involved in the use of parametric models, have led to a strong demand in recent years for the development of non-parametric regression methods. Using these, on the basis of data, the presence of flexible functional forms can be estimated so as to capture possibly complicated relationships between outcomes and covariates. These data-analytical approaches are also referred to as non-parametric techniques [29]. Therefore, the basic principle of non-parametric approaches is to determine the available data structure, most suitable for realizing specific functions, i.e. to determine form of the functions for the available data structure.

Therefore, in the present study, the effects of socio-economic, demographic and geographic factors have been identified using GAMM model with non-parametric age, altitude, total number of rooms, total number of nets, family size and number of months since spraying. In addition to these effects, other parametric effects included in the model were gender, region, main source of drinking water, time taken to collect water, toilet facilities, availability of electricity, radio and television, main material of the dwelling’s walls, roof, and floors, and the use of mosquito nets. In addition to the main effects, four two-way interaction effects were included in the model, namely between gender and main source of drinking water, gender and availability of electricity, gender and main material of the walls of participants’ rooms, and between main source of drinking water and the main material of the rooms’ roof.

The results from the study support those of the previous models fitted. In addition to this, the results give more insight regarding the distribution of age, altitude of participants’ dwellings, total number of rooms, total number of nets, family size and number of months since spraying. The results of the non-parametric part of the model confirm that malaria RDT results are high for children. Moreover, persons with more mosquito nets and a greater number of rooms have better placed to reduce the risk of malaria. By the correct use of mosquito nets, through indoor residual spraying and other preventative measures, like adding more rooms to one’s living quarters, the occurrence of malaria can be reduced. In addition, the study suggests that the poor are less likely to apply these preventative measures and effectively counteract the spread of malaria. The observed association between malaria RDT results and clean drinking water may be explained by the fact that access to clean drinking water is one of the indicators of socio-economic status. Also, maintaining the good condition of a house is essential for controlling the transmission of malaria. Other control measures, including creating awareness about malaria transmission, the use of mosquito nets, and indoor residual sprays, could lead to a reduction in the number of malaria cases.

## Conclusion

In conclusion, the government of Ethiopia has adopted various strategies to the control of malaria. Early diagnosis, prompt treatment, selective vector control, epidemic prevention and control are among the strategies applied by the government. Human resource development, enabling the monitoring and evaluation of malaria, is another supporting strategy to gain control over the disease. It is the government’s key goal to achieve the complete elimination of malaria in those geographical areas with historically a low malaria transmission, and to achieve near-zero transmission of malaria in the remaining areas prone to the disease. To make these goals feasible, policies based on relevant evidence are of the essence [30]. Hence, the results of the present study, showing in detail the extent to which malaria is associated with socio-economic, demographic and geographic factors or, in more general terms, with degrees of poverty, represent a useful contribution to the body of knowledge relevant to the occurrence of malaria in Ethopia. Malaria, generally regarded as a disease that affects the poor plays less of a role in more wealthy households who can afford proper toilet facilities, a well built house with a greater number of rooms, and clean drinking water. Therefore, the observed association between malaria RDT and clean drinking water may be explained by the fact that access to clean drinking water is one of the indicators of socioeconomic status. Poor socio-economic conditions, as the current study demonstrates, are a major contributing factor to the malaria problem. Our results indicate that having more bed nets is one means of reducing its impact. Evidence suggests that households who cannot afford sufficient mosquito nets and who live with large families and low incomes in cramped spaces are vulnerable to malaria. Women and children are exposed to mosquito bites when covering long distances to fetch water. It is obvious that improving the living conditions of communities could be one way of achieving the goals for malaria control, set by the Ethiopian health professionals.

### Ethical clearance

The ethical protocol received approval from the Emory University Institutional Review Board (IRB 1816) and Amhara, Oromiya and SNNPR regional health bureaux. Informed consent was sought in accordance with the tenets of the declaration of Helsinki.

## Competing interests

The authors declare that they have no competing interests.

## Authors’ contributions

DGA acquired the data, performed the analysis and drafted the manuscript. TTZ and HGM designed the research. All authors discussed the results and implications and commented on the manuscript at all stages. All authors contributed extensively to the work presented in this paper. All authors read and approved the final manuscript. All authors contributed extensively to the work presented in this paper.

## Pre-publication history

The pre-publication history for this paper can be accessed here:

http://www.biomedcentral.com/1471-2458/14/31/prepub
